# Is Vaccination a Fraud?

**Published:** 1890-02-08

**Authors:** 


### The Hospital

A Weekly Journal of

Science, Medicine, Nursing, and Philanthropy.

*For Hospitals, Asylums, Medical Practitioners, Students, Nurses, Families, Parishes, Congregations, and the Charitable Public.*

Vol. VII.—No. 176.] [February 8, 1890.

### Is Vaccination a Fraud?

Searching examination, like affliction, is sometimes not pleasant, but grievous; but, also like affliction, it often yields very valuable fruit. The historical criticism of Strauss and Renan caused much tribulation among orthodox theologians; Creighton and Crookshank are making the dry bones of medical dogmatism shake and tremble with the same weapon. No man and no thing need expect in these times to escape the most ruthless investigation. One of the shortest cuts to fame is to attack with boldness and skill cherished institutions. Whilst orthodoxy, though supported by learning, virtue, and the highest ability, may leave its supporters in complete and permanent neglect, heterodoxy, even of a flashy and comparatively unsubstantial kind, brings a man into notice at once. There are thousands of clever young fellows eager to make names for themselves; and nobody knows better than they the difference between the short cut of scientific or religious heterodoxy, and the long, wearisome, and uphill journey of patient and meritorious work. It is not to be wondered at, therefore, that established institutions of every kind should be constant objects of attack and defence.

Many worthy persons shed unavailing tears over this tendency of the human mind to question what is, and to deny its right to continue. That is not the temper of science; it is not the conclusion of reason; it is not the way of progress and development. Man must question, exactly as he must eat, and love, and worship. It is his nature. He has been so made. He can no more resist the impulsion to question and to deny than he can check his tendency to growth in youth and to decay in old age. It is high time, in view of the unparalleled developments of science and the equally extraordinary increase of scientific capacity during the last half-century, that the whole question of vaccination be made the subject of competent re-investigation. Even if compulsory vaccination were not a State institution, the mere fact of the general practice of so peculiar a method of prophylaxy would demand the thoroughest reconsideration at the hands of scientific men. But when every healthy infant in the kingdom is compelled by statute to undergo a surgical operation, and to pass through a critical disease in the interests of the public; and when the warrant for such an enactment dates from what may almost be called pre-scientific times, it is evident to every mind of any critical capacity that the day of judgment for compulsory vaccination has fully come.

The Inquiry Commission which is now sitting on this subject has no holiday task to perform. The friends of compulsory vaccination are numerous and strong. Its enemies are eager, resolute, full of expedients, and have recently received re-inforcement from an unexpected quarter. Professor Creighton was a redoubtable champion; Professor Crookshank, of King's College, promises to be a still more ardent fighter. He has published a book which in point of mere size and price is formidable; whilst as a storehouse of industrious research it is of great value. Professor Crookshank, if his contentions can be maintained, will not only overthrow compulsory vaccination but will destroy the reputation of vaccination itself as a prophylactic. This latter seems a fact of much deeper gravity than the mere question of compulsion. If vaccination is discredited the result will be not merely the destruction of Jenner's reputation, which we all might survive, but also the blasting of the proud fame of the whole medical profession of modern times, and in every civilized country. That is the issue which is really being fought out before the Royal Commission; and it cannot be wondered at that doctors as a class, and especially those of name and high repute, should await with some trepidation the result of the Commission's inquiries.

Professor Crookshank's contentions may be summed up in a few words. He says that "As the result of an investigation into the history, and especially the pathology of vaccination, I feel convinced that the profession has been misled by Jenner, Baron, the reports of the National Vaccine Establishment, and by a want of knowledge concerning the nature of cow-pox, horse-pox and other sources of vaccine lymph. Though in this country vaccine lymph is generally taken to mean the virus of cowpox, yet the pathology of the disease and its nature and affinities have not been made the subject of practical study for nearly half a century. We have submitted instead to purely theoretical teaching, and have been led to regard vaccination as inoculation of the human subject with the virus of a benign disease of the cow, whereas the viruses in use have been derived from several distinct and severe diseases in different animals." We may, perhaps, be permitted to offer a word of explanation here to non-medical readers. It is held as an article of faith by medical men, on the authority of Jenner and others, that if virus taken from cow-pox be employed to inoculate the human subject it will produce a disease, with local sores, which will protect the subject so inoculated against small-pox. It is further held that if lymph be taken from the sores of the human subject, it also has a prophylactic power against small-pox when other human subjects are inoculated by it. The disease produced in the human subject is not held to be true and proper small-pox, but a modified variant of that affection. It is called "vaccinia," and the lymph which may be obtained from the local sores is called "vaccine lymph." This lymph is believed to have had practically but one source, the cow-pox, and to be always and everywhere one and the same virus, attenuated possibly by its transmission through many human systems, but always the lineal descendant of the original cow-pox virus; always the same in essential character, and always acting in the same way as a more or less efficient prophylactic against small-pox.

These are the contentions of scientific and medical orthodoxy. If they are sound, vaccination itself, as a prophylactic, whatever the State may do, has nothing to fear. But are they sound? That is the crux of the whole position. Professor Crookshank maintains that they are not. He says that, regarded as a specific against small-pox, vaccination was historically a failure from the beginning, and pathologically can never, in the nature of things, be anything else to the end. He maintains that whereas cow-pox and small-pox are held to be identical by the orthodox, the actual pathological relationship between the two diseases is really nil. He says, moreover, that mitigation of small pox by vaccine there never was and never can be.

It is evident that nothing can be more thorough-going than Professor Crookshank's conclusions. It is equally obvious that they cannot be adequately traversed at the end of an article. Nor is it desirable, whilst the Royal Commission is still sitting, to attempt a revindication of the orthodox position. But this much at any rate may be advanced at the present moment—that the arguments on the other side will not be wanting either in historic or pathological weight. It may be possible to show that the main stream of vaccine lymph now actually coursing in the veins of tens of thousands of human beings had its origin and source in cow-pox; it may be possible to demonstrate that cow-pox and small-pox, if not identical, are, at least, very near of kin; and it may be possible, by means of unquestionable statistics, to make plain to all men that small-pox has diminished as vaccination has increased, and that the best vaccinated countries are freest from the ravages of that direful disease. It is possible to prove with convincing certainty that the morbid condition resulting from vaccination, and especially that manifestation of it which is evident to the naked eye—namely, the local vesicle or sore which is produced—is practically always the same, alike in its development, in its maturity, and in it decay. If this be indeed so, and if it be true that identical effects must have identical causes, what becomes of the historical demonstration of the variety of sources from which the lymph was originally obtained, and of the diversity of character in the lymph itself? We shall, perhaps, all be well advised to await with patience the result of the inquiries of the Commission which is now sitting, and to enter upon the study of the Commission's Report with open and unprejudiced minds.

